# Combined Treatment of Salinity Stress and Fruit Thinning Effect on Tomato

**DOI:** 10.3389/fnut.2022.857977

**Published:** 2022-03-29

**Authors:** Pengfei Zhang, Shuqin Jiang, Yanyan Dai, Zhaorui Zhang, Masateru Senge

**Affiliations:** ^1^School of Geography Science, Taiyuan Normal University, Jinzhong, China; ^2^Institute for Carbon Neutrality, Taiyuan Normal University, Jinzhong, China; ^3^Eurofins Technology Service (Suzhou) Co., Ltd., Suzhou, China; ^4^Faculty of Applied Biological Sciences, Gifu University, Gifu, Japan

**Keywords:** fruit thinning, fruit quality, growth, logistic equation, salinity stress, water use efficiency, yield

## Abstract

This was an experimental investigation of the combined treatments of salinity (SAL) stress and fruit thinning (FT) on the growth, yield, fruit quality, and water use efficiency (WUE) of tomatoes with non-soil cultivation. The experiment was carried out in a plastic tunnel, Japan. Tomato (*Solanum lycopersicum*) cv. Momotaro seedlings were transplanted in a randomized complete block (RCB) manner with six plants/treatment, and an overall 36 plants in 18 pots (2 plants/pot). The experiment involved varying SAL treatment (no-SAL, moderate SAL, and serious SAL, with electroconductivity of 0.8, 3.0, and 4.5 dS m^−1^, separately) and FT treatment (NT: no thinning and 3FT: three-fruit treatment). The tomato growth, yield, and WUE were significantly suppressed with increasing SAL. In comparison, FT treatment had less effect on tomato growth and water consumption. Either SAL stress or FT treatment significantly improved fruit quality. The combined treatment proved better than single treatment of either SAL stress or FT, avoided the subsize fruit following SAL stress treatment, reduced fruit cracking found with FT treatment, and greatly improved fruit quality. The SAL thresholds of WUEs in relation to biomass, yield, and marketable yield were approximately 3.0 dS m^−1^ under these soilless conditions. Path analysis showed that biomass and water consumption were important indexes affecting yield. Logistic equation fitting showed that SAL stress tended to inhibit and delay plant growth; however, FT tended to advance and shorten the period of plant growth.

## Introduction

Global urbanization was accelerating, and it was reported that 60% of the global population will live in urban areas by 2030 (1). People pay more and more attention to urban food supply and food security (2, 3). As a result, cities around the world are integrating local food production capacity into the built environment to create food security and environmentally sustainable urban agriculture (4). The home garden (also known as household gardens, kitchen gardens, balcony gardens, or homestead gardens) is one form of urban agriculture that represents critical spaces in the configuration of urban socio-ecological landscapes (5, 6). However, the development of home garden was restricted by factors, such as small planting scale, poor professional farming personnel, and low input–output ratio (7).

In recent years, soilless cultivation was used in the home garden to optimize resources and ultimately increase yields (8–10). Soilless culture is broadly adopted to uplift the regulation of the growth environmental conditions and prevent the indeterminacy of the soil (11). It avoids as well the accumulation of salinity (SAL), pests, and illnesses and realizes minimal environmental pollution from irrigation and fertigation (12, 13). Tomato (*Solanum lycopersicum*) is one of the most important vegetable plants in the world. Global production exceeds 180 million metric tons, with China and the USA as the leading producers in 2019, and production in China accounts for 20% of the world (14). Tomato has moderate tolerance to SAL (1.3–6 dS m^−1^) (15), and greater levels of EC reduce yield and fruit size (16, 17) and water and nutrient uptake (18). The negative effects on tomato plant growth are due to SAL stress-causing reductions in root cell growth (19), leaf expansion (20), leaf chlorophyll (21), and plant photosynthesis (22). The decreased output at SAL over the liminal value is due to reductions in the number of fruits produced (19, 23) and in fruit size (24). However, many studies have confirmed that tomato fruit's total soluble solids, titratable acidity, and lycopene, fructose, and glucose concentrations are increased with higher SAL (25, 26).

The studies also showed that yield, fruit quality, and fruit size were affected by numerous reasons, such as plant community (27, 28), fruit thinning (FT) (29, 30), pruning, and variety choice (31). FT is adopted for the limitation of the fruit quantity on each girder and decreases competitive activities to elevate fruit weight. The rise of overall numbers of flowers and fruits can bring more competitive activities for photosynthate and hence decreases fruit weight (29). Abdel-Razzak et al. (32) pointed out that controlling the numbers of flowers, fruits, or fruit girder efficiently decreases competitive activities between fruits, so that extra assimilates are diverted to fewer fruits per girder. FT before harvest decreases fruit total yield per plant (33), but increases fruit size and average fruit weight (32, 34). However, FT was confirmed to accelerate enlargement and increase the size of the retained fruit, thus increasing the risk of fruit cracking and reducing marketable yield (35). In addition, some researchers reported that lower fruit load could increase total soluble solids, VC, titratable acidity, and total carbohydrate of tomato fruit (32).

The environmental thermal status remarkably affects the tomato physiological process (36, 37). Plant growth dynamic models provide the possibility of evaluating the dynamic effects of environmental factors on plant growth. Logistic models are the most commonly used plant dynamic growth simulation models and can be used to depict the association between plant development and thermal unit cumulation (like growing degree days) (38).

According to the above explanation, soilless cultivation is widely used in the home garden because of its convenience and efficiency. SAL stress reduces tomato fruit size and marketable yield, but increases fruit quality. FT increases fruit size and quality, but increases the risk of fruit cracking and so reduces marketable yield. The above two cultivation methods show contradictory advantages and disadvantages for tomato growth. Therefore, it is necessary to determine an appropriate combined treatment of SAL stress and FT for tomatoes. However, the previous research primarily highlighted SAL stress or FT. There has been few considerations of combined treatment, and few relevant studies have used soilless cultivation. Consequently, our research aimed to explore (1) the combined treatment of SAL stress and FT on tomato growth, fruit quality, and WUE under non-soil cultivation and (2) the kinetic features of critical growing indices impacting the plant as per the logistic growing pattern.

## Materials and Methods

### Experiment Site

The assay was carried out inside a plastic tunnel (11 m × 5 m, length and width) at the Experiment Farm of Gifu University, Japan (35°27′51″N, 136°44′14″E). The experiment duration was for 12 weeks from April to July 2018, with average daytime and nighttime temperatures of 28.1 and 20.4°C, respectively, and corresponding average humidity of 51.5 and 79.7%.

### Experimental Design and Treatments

**Figure 1:**
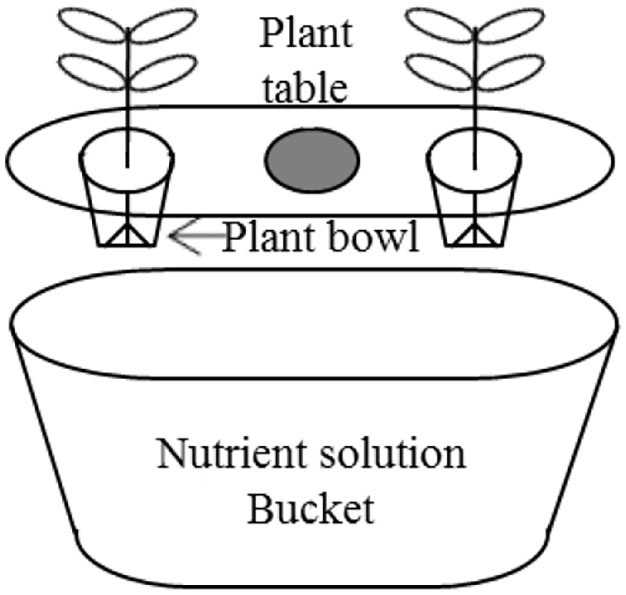
Setup of a hydroponic Power's Pot.

In the experiment, tomato (*Solanum lycopersicum*) cv. Momotaro plants were grown *via* a purchasable hydroponic kit (Minoru Kasei, Co., 388-1 Shimoichi, Japan), which includes a nutrient solution bucket, a plant table, and a plant bowl (Figure 1). The normal cultivation liquid Vegetable Life A (1.3% N, 1.2% P, 1.9% K, 0.32% Mg, 0.008% Mn, and 0.008% B; Otsuka Chemical) was desaturated 200-folds by tap water for the following planting of tomatoes. The desaturated liquor carried an electroconductivity of 0.8 dS m^−1^. NaCl was supplemented into the liquor to bring SAL stress to the plants.

Initially, the seedlings were planted at the above-mentioned farm. If they were nearly 20 cm high, then they would be transplanted in an RCB manner as described in the abstract above. All the pots were full of 14 L of desaturated liquor.

The previous study of our team revealed that the SAL liminal value of tomato registered 1.41 dS m^−1^ *via* the normal cultivation liquid (21). Therefore, SAL levels of 3.0 dS m^−1^ (moderate stress) and 4.5 dS m^−1^ (serious stress) were employed herein. A total of six treatments were utilized, with three electroconductivity results (0.8, 3.0, and 4.5 dS m^−1^) of SAL treatments and two levels (NT: no thinning and 3FT: three fruits) of FT treatments (Table 1). The CN (control treatment) and CF (no stress and 3FT) were the treatments without SAL, MN and MF were the treatments with moderate SAL, and SN and SF were the treatments with serious SAL (Table 1). In addition, CN, MN, and SN were the treatments without FT; CF, MF, and SF were the treatments in which the truss was pruned to have three remaining fruits. Fruits were pruned when they were marble size (31) or when they were at light-maturity stage (32).

**Table 1:** Salinity conditions and fruit thinning for each treatment.

**Treatment**	**Thinning**	**EC (dS m^**−1**^)**	**Description**
CN	No thinning	0.8	Control treatment (no stress-no thinning)
MN		3.0	Moderate stress-no thinning
SN		4.5	Serious stress-no thinning
CF	3-fruits treatment	0.8	No stress-3 fruits
MF		3.0	Moderate stress-3 fruits
SF		4.5	Serious stress-3 fruits

Axillary buds were removed at sprout, and the apical bud was removed in the 5th week (28–35 days posterior to transplantation) when the plant was in the 3rd flower cluster stage. Considering the variation in the climate environment in the house, the location of the pots was changed after irrigation. The cultivation liquid was renewed once every 2 weeks. SAL stress began on May 22 and the first fruits were pruned on May 24.

### Measurements

#### Transpiration

The transpiration (TP, g day^−1^) of each pot was determined *via* gravimetry, with the following calculation:


(1) $$\begin{array}{r} {TP=W_{a}-{W^{\prime }}_{d}+W^{\prime \prime }}_{i} \end{array}$$

in which TP denotes the transpiration when the liquor is renewed (g), W_a_ denotes the weight of the fresh liquor (g), W^′^_d_ denotes the weight of the replaced liquor (g), and W^″^_i_ denotes the weight of watering (g). Watering was carried out supplementing cultivation liquid once every 1 or 2 days. The overall TP of each pot was divided between the two plants in every pot as per their eventual phytomass, and the overall TP of each plant is deemed as the plant's actual TP. The electroconductivity of every pot was surveyed posterior to watering. The solution or NaCl was supplemented when the liquor electroconductivity diverges from the normal values of 3.0 ± 0.2 and 4.5 ± 0.2 dS m^−1^.

#### Plant Growth Parameters

The biomass (g) of every plant was identified posterior to the assay. Leaf chlorophyll was surveyed once a week *via* a SPAD chlorophyll meter (Minolta). A linear association existed between SPAD results and extracted leaf chlorophyll (39, 40); hence, those were employed to reflect the leaf chlorophyll in our study. The dry matter of the superior parts was determined *via* desiccating the plant material at 105°C for half an hour and then at 70°C in the ventilation stove till a steady weight (41).

#### Yield and WUE

Red fruits were harvested two times a week in harvesting periods. The yield (g plant^−1^), the number of flowers, and the number of fruits on every plant were surveyed in harvesting periods. The harvested fruits were divided into marketable or unmarketable. Marketable ones were divided as per size (≥30 mm) and appearance (no cracking). The sizes were measured by vernier caliper. The marketable fruit number and the marketable yield on every plant were also surveyed. The fruit set (%: number of fruits on every plant as a percentage of the total number of flowers) and marketable fruit set (%: number of marketable fruits on every plant as a percentage of the total number of flowers) on every plant were calculated.

The WUE was calculated according to the biomass, yield, and marketable yield, with the following calculations (42):


(2) $$\begin{array}{rcl} WUE_{b} & = & \frac{biomass}{water\;consumption} \end{array}$$

(3) $$\begin{array}{rcl} WUE_{y} & = & \frac{yield}{water\;consumption} \end{array}$$

(4) $$\begin{array}{rcl} WUE_{my} & = & \frac{marketable\;yield}{water\;consumption} \end{array}$$

in which WUE_b_ (g kg^−1^) denotes grams of biomass generated per kg of water; WUE_y_ (g kg^−1^) denotes grams of yield generated per kg of water; and WUE_my_ (g kg^−1^) denotes grams of marketable yield generated per kg of water.

#### Fruit Quality Parameters

Fully matured tomato fruits were used for the fruit quality measurements. A total of eighteen fruits were selected for every treatment, for an overall 108 fruits for the measuring. Fruit firmness was evaluated with a penetrometer. Fruits that showed symptoms of cracking were separately counted to estimate the fruit cracking ratio. The juice of every fruit was extracted *via* a squeezer, and the sugar content (%) and acid content (%) were determined with a Pocket Brix-acid meter (PAL-BX/ACID1, ATAGO Co., Ltd., Tokyo). Moreover, taste index was computed *via* the formula of Hernández-Suárez et al. (43):


(5) $$\begin{array}{r} Taste\;index=\frac{Sugar\;content}{20\;\times \;acidity}+acidity \end{array}$$

### Model Description and Application

#### Path Analysis

Path coefficient analysis divides the components of direct and indirect traits and provides information on direct and indirect effects of interrelated components on yield. Path analysis of plant-growing factors (e.g., root fresh weight, quantity of clusters per plant, individual fruit weight, number of fruits per plant, and chlorophyll stability index) and the yield not only displays the correlation between these factors, but also presents the direct and indirect effects of growth factors on yield (38, 44). In this research, biomass (X1), plant height (X2), leaf chlorophyll (X3), and water consumption (X4) as independent variates and yield (Y) as the dependent variate were selected to calculate path coefficients.

#### Logistic Equation

A logistic equation was used to quantify the effects of SAL and FT on the growth of tomatoes, which is expressed below (38, 45):


(6) $$\begin{array}{r} y=\frac{k}{\left(1+a\cdot EXP\left(b\cdot t\right)\right)} \end{array}$$

in which *y* denotes the dependent growing parameter or plant biomass cumulation (g plant^−1^); *k* denotes the top asymptotic line reflecting the superior limit of plant growth; *a* and *b* denote quotient of the preliminary stage and accretive rate, separately; and *t* denotes the independent runtime (°C d).

Feature parameters of the logistic equation were computed below (45):


(7) $$\begin{array}{rcl} v_{m} & = & \frac{bk}{4} \end{array}$$

(8) $$\begin{array}{rcl} t_{m} & = & \frac{lna}{b} \end{array}$$

(9) $$\begin{array}{rcl} t_{1} & = & \frac{1}{b}\ln\left(\frac{2+\sqrt{3}}{a}\right) \end{array}$$

(10) $$\begin{array}{rcl} t_{2} & = & \frac{1}{b}\ln\left(\frac{2-\sqrt{3}}{a}\right) \end{array}$$

(11) $$\begin{array}{rcl} t_{d} & = & t_{2}-t_{1} \end{array}$$

where *v*_*m*_ (g plant^−1^ °C^−1^ d^−1^ for plant biomass cumulation) is the maximal slope and is deemed as the maximal growing velocity in the fast-growing stage; *t*_*m*_ (°C d) denotes the relevant time of *v*_*m*_ and denotes the time when growing velocity registers its maximal value; *t*_1_ and *t*_2_ (°C d) denote the inflexion of the logistic equation and represent the nodal points of the start and end time of the fast-growing period, respectively; and *t*_*d*_ denotes the duration of the fast-growing period (°C d).

Accumulated thermal unit is adopted in the experiment farm with a small meteorological station, rather than calendar days posterior to cultivation. The formula adopted to calculate thermal time stands thus:


(12) $$\begin{array}{r} GDD=\Sigma \frac{T_{\max}+T_{\min}}{2}-T_{base} \end{array}$$

in which GDD is the growing degree days; *T*_*max*_ and *T*_*min*_ denote the daily maximal and minimal temperature (°C), separately; and *T*_*base*_ denotes the basic temperature of 10°C for greenhouse tomato (38).

### Data Analysis

Statistical analysis was carried out *via* two-way analysis of variance, and the results were afterward in contrast to Duncan's test with a credit level of 5% *via* the R program language. The logistic equation was fitted using Origin 2019b.

## Results

### Plant Growth

Plant biomass, dry matter, and chlorophyll significantly reduced with increasing SAL regardless of thinning condition (Table 2). The plant growth variables exhibited no significant differences with varied FT conditions (Table 2). The biomass and dry matter were no significant differences with combined treatment (SAL and FT), but leaf chlorophyll was significantly different. Meanwhile, the values of growth variables under MF and SF treatments (combined treatment) were remarkably smaller vs. those under the CN.

**Table 2:** Combined treatment on biomass, dry matter, and leaf chlorophyll (SPAD value).

**Treatment**		**Biomass (g)**		**Dry matter**		**Chlorophyll**	
CN		2,115.7 ± 252.7		129 ± 14.9		52.9 ± 1.7	
MN		1,359.9 ± 55.2		58.5 ± 6.4		51.0 ± 1.8	
SN		917.5 ± 225.3		53.0 ± 7.0		50.9 ± 0.8	
CF		1,954.3 ± 83.4		116.5 ± 7.2		53.8 ± 1.6	
MF		1,325.2 ± 74.5		67.1 ± 9.8		51.0 ± 0.9	
SF		1,004.6 ± 98.3		48.5 ± 6.4		48.5 ± 0.9	
SAL (*n* = 12)	0.8	2,035.0 ± 198.2	a	122.8 ± 11.7	a	52.4 ± 1.6	a
	3.0	1,342.5 ± 65.1	b	62.8 ± 7.7	b	51.0 ± 1.4	b
	4.5	961.0 ± 171.8	c	50.8 ± 6.7	c	49.7 ± 1.5	c
FT (*n* = 18)	NT	1,464.4 ± 541.9	a	80.2 ± 14.8	a	51.6 ± 1.7	a
	3FT	1,428.0 ± 413.9	a	77.4 ± 10.3	a	51.1 ± 2.5	a
SAL*FT		NS		NS		S	

*Data are means ± SD*.

*Values followed by different small letters (a–c) in the same column significantly differ (P < 0.05) by Duncan's test*.

### Yield

Yield variables significantly decreased with increasing SAL. Compared to NT, the 3FT treatment showed significantly lower fruit number, fruit set, marketable fruit number, and marketable fruit set. For combined treatment, these yield variables showed no evident differences except marketable yield (Table 3).

**Table 3:** Combined treatment on flower number, fruit number, fruit set, marketable fruit number, marketable fruit set, yield, and marketable yield.

**Treatment**		**Flower NO**. **(plant**^**−1**^**)**		**Fruit NO**. **(plant**^**−1**^**)**		**Fruit set** **(%)**		**Marketable fruit NO**. **(plant**^**−1**^**)**		**Marketable fruit set** **(%)**		**Yield** **(g plant**^**−1**^**)**		**Marketable yield** **(g plant**^**−1**^**)**	
		**②**		**③**		**③/**②		**④**		**④** **/** **②**		**⑤**		**⑥**	
CN		16.0 ± 1.3		11.5 ± 1.0		72.3 ± 9.2		8.7 ± 2.3		54.4 ± 15.5		964.3 ± 201.8		770.0 ± 161.2	
MN		13.8 ± 1.2		7.5 ± 2.8		54.0 ± 19.2		5.7 ± 2.3		40.7 ± 15.4		599.4 ± 41.4		422.9 ± 37.5	
SN		13.8 ± 0.8		5.7 ± 1.8		40.3 ± 6.9		3.7 ± 1.4		26.9 ± 10.9		270.5 ± 169.7		218.4 ± 137.1	
CF		15.7 ± 1.4		9.0 ± 1.1		57.7 ± 7.3		6.0 ± 1.3		38.5 ± 8.9		811.0 ± 104.2		513.8 ± 66.0	
MF		14.3 ± 1.6		6.2 ± 0.4		43.7 ± 7.9		4.3 ± 1.0		30.5 ± 7.8		498.5 ± 61.2		435.9 ± 47.3	
SF		13.7 ± 0.8		5.3 ± 1.6		39.4 ± 13.1		3.5 ± 1.4		25.9 ± 11.1		313.7 ± 81.7		265.8 ± 69.2	
SAL (*n* = 12)	0.8	15.8 ± 1.3	a	10.3 ± 1.7	a	65.0 ± 11.0	a	7.3 ± 2.3	a	46.5 ± 14.6	a	887.7 ± 172.8	a	641.9 ± 178.0	a
	3.0	14.0 ± 1.4	b	6.8 ± 2.0	b	48.9 ± 15.0	b	5.0 ± 1.9	b	35.6 ± 12.8	b	549.0 ± 72.5	b	429.4 ± 41.3	b
	4.5	13.8 ± 0.8	b	5.5 ± 1.6	b	39.8 ± 10.0	b	3.6 ± 1.3	b	26.4 ± 10.5	b	292.1 ± 129.0	c	242.1 ± 106.4	c
FT (*n* = 18)	NT	14.6 ± 1.5	a	8.2 ± 3.1	a	55.5 ± 18.2	a	6.0 ± 2.8	a	40.6 ± 17.6	a	611.4 ± 325.6	a	470.5 ± 261.7	a
	3FT	14.6 ± 1.5	a	6.8 ± 1.9	b	46.9 ± 12.2	b	4.6 ± 1.5	b	31.7 ± 10.3	b	541.1 ± 225.5	a	405.2 ± 121.2	a
SAL*FT		NS		NS		NS		NS		NS		NS		S	

*Data are means ± SD*.

*Values followed by different small letters (a–c) in the same column significantly differ (P < 0.05) by Duncan's test*.

### WUE

**Figure 2:**
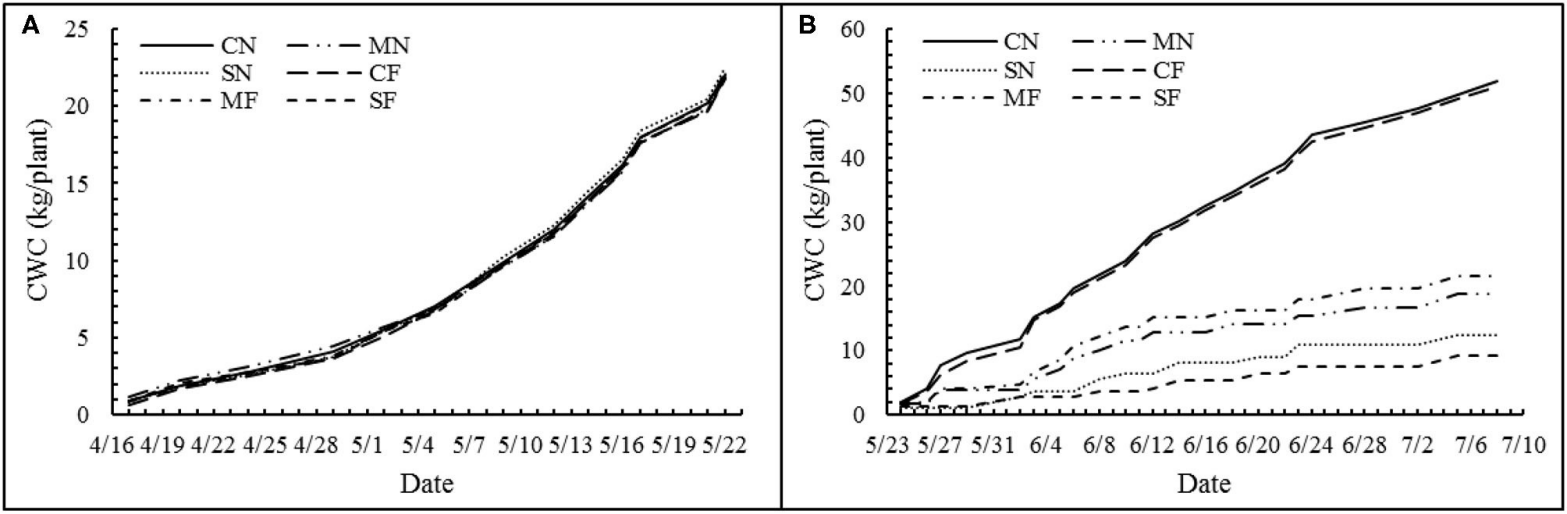
Cumulative water consumption (CWC) of each treatment before **(A)** and after **(B)** salinity stress.

In this study, the daily plant's actual TP of a plant was defined as the daily water consumption. Meanwhile, for the convenience of comparison with soil cultivation, the daily water consumption of a plant was expressed as “mm.” The cumulative water consumption (CWC) before and after SAL treatment is shown in Figures 2A,B, respectively. The CWC did not differ among the treatments before applying SAL (Figure 2A) but significantly decreased with increasing SAL (Figure 2B). However, the CWC was not different among the same SAL conditions (i.e., CN and CF; MN and MF; and SN and SF) (Figure 2B). The WUE for biomass, yield, and marketable yield showed an initial rise and a subsequent reduction with increasing SAL. The WUE variables exhibited no significant differences with varied FT conditions. For combined treatment, these WUE variables showed significant differences (Table 4).

**Table 4:** Combined treatment on cultivation water consumption (CWC), water use efficiency for biomass (WUE_b_), water use efficiency for yield (WUE_y_), and water use efficiency for marketable yield (WUE_my_).

**Treatment**		**Water Con. (kg)**		**WUE**_**b**_ **(g kg**^**−1**^**)**		**WUE**_**y**_ **(g kg**^**−1**^**)**		**WUE**_**my**_ **(g kg**^**−1**^**)**	
		**⑦**		^①*^ **1,000/** **⑦**		⑤*1,000/⑦		**⑥** *1,000/ **⑦**	
CN		74.0 ± 8.6		28.6 ± 0.5		12.9 ± 1.3		10.3 ± 1.1	
MN		40.7 ± 1.9		33.5 ± 0.9		14.8 ± 1.3		10.4 ± 1.1	
SN		35.0 ± 2.2		26.2 ± 5.8		7.7 ± 4.7		6.2 ± 3.8	
CF		72.8 ± 2.1		26.8 ± 1.0		11.1 ± 1.2		7.0 ± 0.8	
MF		43.5 ± 2.5		30.5 ± 0.6		11.4 ± 1.0		10.0 ± 0.7	
SF		31.3 ± 2.6		32.1 ± 1.7		9.9 ± 1.8		8.4 ± 1.5	
SAL (*n* = 12)	0.8	73.4 ± 6.0	a	27.7 ± 1.2	b	12.0 ± 1.5	a	8.7 ± 1.9	ab
	3.0	42.1 ± 2.5	b	32.0 ± 1.7	a	13.1 ± 2.1	a	10.2 ± 0.9	a
	4.5	33.1 ± 3.0	c	29.1 ± 5.1	b	8.8 ± 3.6	b	7.3 ± 2.9	b
FT (*n* = 18)	NT	49.9 ± 18.4	a	29.4 ± 4.5	a	11.8 ± 4.1	a	9.0 ± 3.0	a
	3FT	49.2 ± 18.1	a	29.8 ± 2.5	a	10.8 ± 1.5	a	8.5 ± 1.6	a
SAL*FT		NS		S		S		S	

*Data are means ± SD*.

*Values followed by different small letters (a–c) in the same column significantly differ (P < 0.05) by Duncan's test*.

### Fruit Quality

The parameters for fruit quality (sugar, acid, and taste index) were significantly enhanced with increasing SAL (Table 5). The values of fruit quality variables under 3FT treatment were significantly higher than for NT treatment. Notably, the sugar, acid, and taste index were higher under 3FT than NT treatment by 7.9, 12.5, and 10.1%, respectively. Fruit firmness decreased with increasing SAL, and FT resulted in significant softening of the fruit. The SAL stress significantly decreased the fruit cracking ratio, and FT significantly increased it. In addition, for combined treatment, despite no evident differences, the fruit quality variables under MF and SF treatments (combined treatment) showed the highest values among the treatments. The fruit firmness showed no evident differences with combined treatment, and the fruit cracking ratio was significantly different.

**Table 5:** Combined treatment on fruit quality.

**Treatment**		**Sugar (%)**		**Acid (%)**		**Taste index**		**Fruit firmness**		**Cracking ratio (%)**	
CN		4.44 ± 0.61		0.79 ± 0.08		1.07 ± 0.09		1.08 ± 0.13		17.9 ± 2.0	
MN		8.40 ± 0.49		1.73 ± 0.10		1.97 ± 0.10		0.99 ± 0.13		0.0 ± 0.0	
SN		9.74 ± 0.57		2.05 ± 0.17		2.29 ± 0.16		0.89 ± 0.21		0.0 ± 0.0	
CF		4.83 ± 0.28		0.84 ± 0.05		1.13 ± 0.03		0.77 ± 0.14		30.6 ± 12.4	
MF		9.41 ± 0.25		2.18 ± 0.41		2.40 ± 0.37		0.75 ± 0.14		22.4 ± 8.0	
SF		10.12 ± 0.97		2.12 ± 0.34		2.36 ± 0.32		0.84 ± 0.12		0.0 ± 0.0	
SAL (*n* = 12)	0.8	4.64 ± 0.50	c	0.82 ± 0.07	b	1.10 ± 0.07	b	0.93 ± 0.13	a	21.4 ± 15.4	a
	3.0	8.91 ± 0.65	b	1.95 ± 0.37	a	2.19 ± 0.34	a	0.87 ± 0.14	b	11.0 ± 9.8	b
	4.5	9.93 ± 0.78	a	2.08 ± 0.26	a	2.32 ± 0.25	a	0.87 ± 0.17	b	0.0 ± 0.0	c
FT (*n* = 18)	NT	7.52 ± 2.38	b	1.52 ± 0.56	b	1.78 ± 0.54	c	0.99 ± 0.15	a	6.0 ± 4.7	b
	3FT	8.12 ± 2.48	a	1.71 ± 0.70	a	1.96 ± 0.66	a	0.79 ± 0.13	b	17.7 ± 8.9	a
SAL*FT		NS		NS		NS		NS		S	

*Data are means ± SD*.

*Values followed by different small letters (a–c) in the same column significantly differ (P < 0.05) by Duncan's test*.

### Path and Logistic Analyses

Yield showed the greatest value of variable coefficient (47.6%), followed by water consumption (35.7%) and biomass (32.4%), with the lowest value for plant height (4.0%) (Table 6). The *F*-values between plant growth indicators (biomass, plant height, leaf chlorophyll, and water consumption) and yield showed very significant correlations and indicated that regression analysis could be performed (Table 6).

**Table 6:** The decomposition of the simple correlation coefficient and analysis of variance of agronomic parameters.

**Argument**	**Path analysis**				**Simple correlation coefficient with yield (R^**2**^)**	**Variance analysis with yield**		
	**X1**	**X2**	**X3**	**X4**		**Standard deviation**	**Variable coefficient/%**	***F* value**
Biomass (X1)	1.523	0.016	0.039	−0.61	0.933	468.96	32.4	89.72**
Height (X2)	−0.24	–0.1	−0.01	0.171	0.03	3.68	4	128.42**
Chlorophyll (X3)	0.959	0.008	0.063	−0.45	0.339	2.09	4.1	128.05**
Water consumption (X4)	1.462	0.028	0.044	–0.63	0.813	17.7	35.7	128.42**
Yield	–	–	–	—	—	274.41	47.6	—

Values marked by “underline” are direct effects. “**” most significantly different (P < 0.01).

Path analysis showed that biomass had the strongest effect on yield, mainly based on the direct (1.523) and indirect effects of water consumption (−0.609) (Table 6). Water consumption showed the second greatest effect on yield mainly based on direct (−0.634) and indirect effects of biomass (1.462). This was followed by plant height, mainly based on the direct (−0.103) and indirect effects of biomass (−0.244) and indirect effect of water consumption (0.171). Chlorophyll had the lowest effect on yield mainly based on the indirect effects of biomass (0.959) and water consumption (−0.45). The direct effect of water consumption on yield had the largest negative value (−0.634), and its correlation coefficient had a larger positive value (0.813). The above explanation indicated that the indicators of biomass and water consumption were the most relevant to yield.

**Figure 3:**
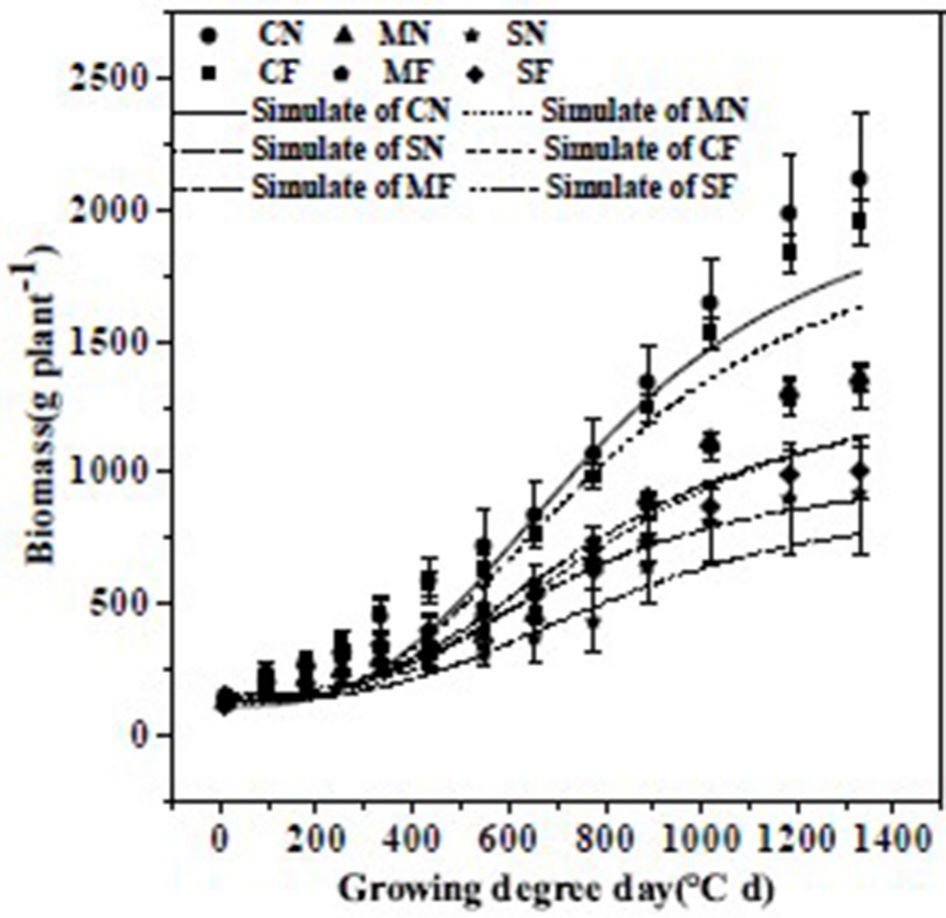
Comparisons between the measured and simulated values (using the logistic equation) of biomass under different salinity stress and fruit thinning. Bars are means ± standard deviation (*n* = 6). Note: Trend line formulas are: CN, *y* = 2,450/(1 + 16.18038975 × exp(−0.0035x)); MN, y = 2,279/(1 + 17.29815762 × exp(−0.0026x)); SN, y = 3,614/(1 + 24.83366022 × exp(−0.0017x)); CF, y = 2,322/(1 + 14.56760168 × exp(−0.0033x)); MF, y = 2,341/(1 + 10.96929493 × exp(−0.0025x)); SF, y = 1,224/(1 + 6.685894442 × exp(−0.0027x)).

The path analysis showed the significance of biomass on yield. As the logistic equation performed well in the kinetic description of plant growth with the parameters, it was fitted to the association between accumulated temperature and kinetic variations in tomato biomass. Biomass for diverse SAL and FT treatments was fitted *via* logistic equations (Figure 3). The root mean square error range was 26.47–60.44 g plant^−1^ with a mean of 49.01 g plant^−1^, the index of agreement (d) range was 0.09–0.82 with an average of 0.56, and coefficients of determination (R^2^) were 0.977–0.994 with an average of 0.987.

The values of *t*_1_, *t*_2_, and *t*_*m*_ rose with increasing SAL (Table 7). The start of the rapid growth period (*t*_1_) for S_3.0_ and S_4.5_ was 6 and 12 days later than for S_0.8_, respectively, with a slope of 3.2 days (dS m^−1^)^−1^ (R^2^ = 0.988). The end of the rapid growth period (*t*_2_) of S_3.0_ and S_4.5_ was 4 and 28 days later than S_0.8_, respectively, with a slope of 6.39 days (dS m^−1^)^−1^ (R^2^ = 0.784). The periods of most rapid growth (*t*_*m*_) of S_3.0_ and S_4.5_ were 10 and 20 days later than S_0.8_, respectively, with a slope of 5.06 days (dS m^−1^)^−1^ (R^2^ = 0.981). The maximal growing velocity in the fast-growing stage (*v*_*m*_) dropped with increasing SAL.

**Table 7:** Characteristic parameters calculated by logistic equation for biomass accumulation among the treatments.

**Treatment**	**Characteristic parameters**				
	**t_**1**_ (**°**C d)**	**t_**2**_ (**°**C d)**	**t_**m**_ (**°**C d)**	**t_**d**_ (**°**C d)**	**v_**m**_ [g (plant **°**C d)^**−1**^]**
CN	419.1	1,171.6	795.4	752.5	2.14
MN	589.9	1,602.9	1,096.4	1,013	1.48
SN	1,114.8	2,664.2	1,889.5	1,549.4	1.54
CF	412.7	1,210.8	811.8	798.2	1.92
MF	431.3	1,484.8	958	1,053.6	1.18
SF	215.9	1,191.5	703.7	975.5	0.83
S_0.8_	415.9	1,191.2	803.6	775.4	2.03
S_3.0_	510.6	1,543.9	1,027.2	1,033.3	1.33
S_4.5_	665.4	1,927.8	1,296.6	1,262.4	1.18
NT	707.9	1,812.9	1,260.4	1,105	1.72
3FT	353.3	1,295.7	824.5	942.4	1.31

*S_0.8_, S_3.0_, and S_4.5_ are no-salinity, moderate salinity, and serious salinity, respectively; NT and 3FT are no-thinning and three-fruit treatment, respectively*.

## Discussion

The tomato plant growth variables decreased with increasing SAL (Table 2). The decrease of biomass and dry matter is likely because SAL stress restrained plant organ (root, shoot, and leaf) development (20, 46). Albacete et al. (47) showed that the fresh weight of tomato root was reduced by 30% posterior to 3 weeks under SAL conditions (100 mM NaCl). Kamrani et al. (20) found that SAL up to 20 mM affected tomato shoot development, and that elevated SAL significantly decreased seedling height. Oztekin and Tuzel (48) discovered that the mean seedling height of 21 purchasable tomato cultivars was reduced by 29.03% under 200 mM NaCl treatment in contrast to non-SAL treatment. Azarmi et al. (49) revealed that the overall foliage area dropped with rising SAL (EC: 2.5–6 dS m^−1^). The decrease of leaf chlorophyll content was associated with salt-caused increase in chlorophyllase activity, which casted an unfavorable effect on membranous stability and attenuation of protein–colorant–lipin complex (50). The decrease in leaf chlorophyll was consistent with the results of Azarmi et al. (49), which shows that leaf chlorophyll content was decreased with SAL.

Plant growth variables did not significantly differ with varied FT conditions, which indicates that FT did not cast impact casted on the plant growth. Tomato leaves, stems, and roots are the prime source organs for assimilates, so FT had no effect on assimilate products. Although FT may reduce the consumption of assimilating products, the excess may be absorbed by other plant organs (51). In conclusion, FT did not affect the growth of tomato plants.

Although combined treatment (MF and SF) of SAL stress and FT remarkably reduced the biomass, dry matter, and chlorophyll, no difference was evident with varied FT conditions. This indicated that tomato growth variables were reduced due to SAL stress but not FT.

Tomato yield variables significantly decreased with increasing SAL. This conclusion was consistent with many other studies. Qaryouti et al. (52) discovered that the overall yield of tomato cv. Durinta F1 was remarkably decreased at SAL of 5 dS m^−1^ and above, with a 7.2% yield decrease per unit rise in SAL. Moreover, Magan et al. (53) discovered that tomato overall and marketable yield dropped remarkably with rising SAL. At present, although there are different opinions concerning how SAL stress affects tomato yield, many researchers believe that fruit size is remarkably dropped with rising SAL (54, 55).

Most yield variables were significantly lower for 3FT than for NT treatment. This could be explained by lower values of fruit number and marketable fruit number for 3FT treatment, because only three fruits remained for each cluster. Therefore, the fruit set and marketable fruit set were lower than in the NT treatment. However, yield under SF and marketable yield under MF and SF (combined treatment) were slightly higher than CN condition. This result indicated that the FT method (3FT) in this study failed to achieve the threshold for marketable yield. We may attempt in subsequent experiments to retain four or more fruits to maximize marketable yield. This result also confirms the different effects of SAL stress and FT on tomato yield and marketable yield. Salinity stress reduces yield and marketable yield, whereas reasonable FT can increase yield and marketable yield by increasing weight per fruit. The values of yield variables also exhibited slight differences under the same SAL condition (e.g., MN and MF, and SN and SF). This result further elucidates that the loss of tomato yield due to SAL stress can be compensated by reducing fruit load and removing substandard fruit in advance, thus achieving no reduction in yield or even an increase in marketable yield. In other words, proper FT can alleviate the effect of SAL stress on tomato yield.

The discrepancies in CWC before and after applying SAL were caused by SAL stress rather than FT. Zhang et al. (22) showed that SAL would reduce root water absorption *via* the osmosis effect and afterward induces water stress. Ahanger et al. (18) indicated as well that plant water uptake is reduced with increased SAL. SAL stress disrupts the osmosis balance and causes dropping water absorption and closing of stoma aperture, which leads to the suppression of evapotranspiration (56).

The daily water consumption was 0.92 × 10^−3^ mm plant^−1^ before applying SAL. The daily water consumption after applying SAL ranged from 0.29 × 10^−3^ to 1.62 × 10^−3^ mm plant^−1^ with a mean of 0.86 × 10^−3^ mm plant^−1^. During the whole growth period, daily water consumption ranged from 0.55 × 10^−3^ to 1.31 × 10^−3^ mm plant^−1^ with a mean of 0.87 × 10^−3^ mm plant^−1^. Wang et al. (57) showed that daily water consumption ranged from 71.6 × 10^−3^ to 91.9 × 10^−3^ mm plant^−1^ under greenhouse soil cultivation. The daily water consumption of tomatoes under soilless cultivation conditions was significantly lower than that found for soil cultivation environments, although comparability of the results might be affected by such differences as cultivation environment and cultivar.

The WUE variables showed an initial rise and a subsequent reduction with increasing SAL (Table 4). The SAL interval used in this study was not conducive to evaluating the SAL threshold due to its large range; however, it could be approximately determined that the SAL thresholds of WUE variables were nearly 3.0 dS m^−1^ under soilless conditions using Vegetable Life A nutrient solution.

It is a common practice in commercial tomato production to add salt to nutrient solutions to improve fruit quality. Many experiments have indicated that SAL stress can increase tomato quality fruit (19). The 3FT treatment showed higher fruit quality. It may be because tomato plant growth is highly dependent on source, sink strength, and their equilibrium (58). Leaves are both source and sink organs. However, fruit is the strongest sink for assimilate. Therefore, FT treatment will not decrease assimilation substance generation rate, whereas it will decrease the competitive activities for assimilate among fruits (59). The highest values of fruit quality variables were for MF and SF treatments because both SAL stress and FT improved tomato fruit quality, and their dual effects could greatly improve fruit quality.

Fruit cracking is affected by the cultivar, fruit size, fruit firmness, fruit shape, fruit growing, fruit cuticle and saccharinity, irrigation water quality, and environmental conditions (35). Among these factors, firmness has a strong influence on cracking (60). The fruit firmness and fruit cracking ratio decreased with increasing SAL in this study, likely because tomato skin becomes tougher and more resistant to tearing under saline conditions (60). Krauss et al. (61) showed that fruit firmness decreased with increasing SAL. However, most studies have reported increased (62) or unchanged (63) fruit firmness under saline conditions. The differences may result from the use of different cultivars and periods of fruit picking. In addition, lower fruit firmness mitigates fruit cracking due to enhanced cross-linking of pectin in the pericarp cells (64).

The results of path and logistic analyses indicated that the SAL stress tended to inhibit and delay plant growth. Zhang et al. (22) showed that SAL inhibits tomato plant growth by negatively affecting tomato roots, shoots, leaves, yield, and WUE.

The *t*_1_, *t*_2_, and *t*_*m*_ values under three-fruit treatment were earlier than for the no-thinning treatment (Table 7), by 17, 19, and 18 days, respectively. The *v*_*m*_ under the three-fruit treatment was also lower than for the no-thinning treatment.

Thus, FT tended to advance and shorten the period of plant growth, possibly because the lower fruit load decreased the competition for assimilating (e.g., water, nutrients, and carbon) between the fruits and plant growth (65).

## Conclusions

The experimental results showed that tomato growth variables were significantly decreased with increasing SAL, but FT treatment had less effect on tomato growth.

Salinity stress was confirmed to improve fruit quality and reduce fruit cracking, but reduced marketable yield by reducing fruit size. FT was confirmed to improve fruit quality and marketable yield by removing unhealthy fruit and increasing remaining fruit size, but increasing fruit cracking. Combined treatment of SAL stress and FT avoided the problem of subsize fruit under SAL stress treatment, reduced the fruit cracking found with FT treatment, and greatly improved marketable yield and fruit quality.

Salinity stress suppressed water absorption of tomato, but FT had no effect on water consumption. The daily water consumption before SAL treatment, after treatment, and for the whole growth period was 0.92 × 10^−3^, 0.86 × 10^−3^, and 0.87 × 10^−3^ mm plant^−1^, respectively. The daily water consumption of tomatoes was significantly lower for soilless than for soil cultivation. The SAL thresholds of WUE variables were determined to be approximately 3.0 dS m^−1^ under soilless conditions using Vegetable Life A nutrient solution.

Path analysis revealed that biomass and water consumption were vital indices positively influencing yield. Based on the logistic equation fitting, the SAL stress tended to inhibit and delay plant growth. The rapid growth period (*t*_1_), end of the rapid growth period (*t*_2_), and most rapid growth time (*t*_*m*_) and SAL stress showed that increasing SAL by 1 dS m^−1^ resulted in 3.20, 6.39, and 5.06 days of delay, respectively. FT tended to advance and shorten the period of plant growth.

## Data Availability Statement

The original contributions presented in the study are included in the article/supplementary materials, further inquiries can be directed to the corresponding author/s.

## Author Contributions

PZ, SJ, YD, and MS: conceptualization. PZ, SJ, ZZ, and YD: writing-original draft. PZ, YD, and MS: writing, reviewing, and editing. All authors contributed to the article and approved the submitted version.

## Funding

This study was supported by Shanxi Scholarship Council of China (2020-138), Education Department of Shanxi Province (2019L0781 and 2021L436), and Shanxi Federation of Social Sciences (2019B375 and 2020YY207).

## Conflict of Interest

SJ was employed by Eurofins Technology Service (Suzhou) Co., Ltd. The remaining authors declare that the research was conducted in the absence of any commercial or financial relationships that could be construed as a potential conflict of interest.

## Publisher's Note

All claims expressed in this article are solely those of the authors and do not necessarily represent those of their affiliated organizations, or those of the publisher, the editors and the reviewers. Any product that may be evaluated in this article, or claim that may be made by its manufacturer, is not guaranteed or endorsed by the publisher.
